# GFAP expression and social deficits in transgenic mice overexpressing human sAPPα

**DOI:** 10.1002/glia.22544

**Published:** 2013-07-10

**Authors:** Antoinette R Bailey, Huayan Hou, Min Song, Demian F Obregon, Samantha Portis, Steven Barger, Doug Shytle, Saundra Stock, Takashi Mori, Paul G Sanberg, Tanya Murphy, Jun Tan

**Affiliations:** 1 Department of Psychiatry and Neurosciences, Rashid Laboratory for Developmental Neurobiology, Morsani College of Medicine, University of South FloridaTampa, Florida; 2 Department of Psychiatry and Neurosciences, Silver Child Development Center, Morsani College of Medicine, University of South FloridaTampa, Florida; 3 Department of Geriatrics, University of Arkansas for Medical SciencesLittle Rock, Arkansas; 4 Department of Neurosurgery and Brain Repair; Center for Aging and Brain Repair, Morsani College of Medicine, University of South FloridaTampa, Florida; 5 Department of Biomedical Sciences, Saitama Medical Center and UniversityKawagoe, Saitama, Japan; 6 Department of Pathology, Saitama Medical Center and UniversityKawagoe, Saitama, Japan; 7 Department of Pediatrics, Rothman Center for Neuropsychiatry, Morsani College of Medicine, University of South Florida, All Children’s HospitalSt. Petersburg, Florida

**Keywords:** sAPPα, astrogliosis, autism, behavior, IL-6, Notch

## Abstract

Autistic individuals display impaired social interactions and language, and restricted, stereotyped behaviors. Elevated levels of secreted amyloid precursor protein-alpha (sAPPα), the product of α-secretase cleavage of APP, are found in the plasma of some individuals with autism. The sAPPα protein is neurotrophic and neuroprotective and recently showed a correlation to glial differentiation in human neural stem cells (NSCs) via the IL-6 pathway. Considering evidence of gliosis in postmortem autistic brains, we hypothesized that subsets of patients with autism would exhibit elevations in CNS sAPPα and mice generated to mimic this observation would display markers suggestive of gliosis and autism-like behavior. Elevations in sAPPα levels were observed in brains of autistic patients compared to controls. Transgenic mice engineered to overexpress human sAPPα (TgsAPPα mice) displayed hypoactivity, impaired sociability, increased brain glial fibrillary acidic protein (GFAP) expression, and altered Notch1 and IL-6 levels. NSCs isolated from TgsAPPα mice, and those derived from wild-type mice treated with sAPPα, displayed suppressed β-tubulin III and elevated GFAP expression. These results suggest that elevations in brain sAPPα levels are observed in subsets of individuals with autism and TgsAPPα mice display signs suggestive of gliosis and behavioral impairment.

## Introduction

Autism is a heterogeneous neurodevelopmental disorder characterized by impaired communication, social interaction, and restricted, repetitive behaviors and interests (APA, 3; Steyaert and De la Marche, 2008). Patients with autism exhibit varied patterns of aberrant neuroanatomical and immunological features in addition to behavioral phenotypes (Bailey et al., 4; Bauman and Kemper, 10; Casanova et al., 15; Amaral et al., 2). Brain overgrowth followed by decelerated development is frequently observed in some autistic patients. This neuropathology is observed early in the developmental course and is thought to involve defects in the normal “pruning” of early neural network arbors. After proliferation and dendritic arbor formation, pruning and programmed cell death are highly regulated multifactorial processes dependent on ordered molecular and cellular interactions involving various players including neural stem cells (NSCs), astrocytes, and microglia.

Typically, glial cells are generated after neurogenesis in the CNS (Sofroniew and Vinters 60). The full complement of neurons appears during the embryonic period while most gliogenesis occurs within the first month after birth (Jacobson, 27). Besides this sequential schedule of NSC differentiation, astrogliogenesis can be prematurely induced. In murine embryonic Day 15 (E15) NSC cultures, treatment with IL-6 family proteins that stimulate glycoprotein 130 (gp130) and activate the Janus kinase-signal transducers and activators of transcription (JAK/STAT) pathway (Marz et al., 42; Taga and Fukuda, 66), led to the upregulation of the astrocyte marker glial fibrillary acidic protein (GFAP) (Bonni et al., 13; Deverman and Patterson, 20; Kirsch et al., 29).

The gp130 pathway cooperates with the Notch1 pathway, which also enhances NSC commitment to glial fate by suppressing neuronal differentiation (Bhattacharya et al., 11; Sugaya, 64; Rodriguez-Rivera et al., 55). There is also evidence of crosstalk between the Notch1 and gp130 pathways as increased Hairy and enhancer of split (Hes), resulting from Notch1 activation, promotes phosphorylation of signal transducer and activator of transcription 3 (STAT3), a transcription factor for GFAP (Grandbarbe et al., 26; Nagao et al., 49).

Given that astrocytes are important for synaptic pruning during development, promotion of astrocytic differentiation and abnormal glial activation or gliosis appears sufficient to affect neurodevelopment (Oland and Tolbert, 50; Stephan et al., 62). Several studies give evidence for gliosis in different brain regions of autistic patients (Ahlsen et al., 1; Sabaratnam, 56; Laurence and Fatemi, 38; Vargas et al., 68). How abnormal cellular, inflammatory, and neurotropic signals align leading to excessive, aberrant dendritic connectivity via impaired pruning in autistic individuals is still unclear.

The amyloid precursor protein (APP) is a single-pass transmembrane glycoprotein consisting of 695–770 amino acids and existing in three main isoforms (Selkoe et al., 58; Turner et al., 67). APP is processed according to two separate pathways that produce different protein fragments. Protein processing by the amyloidogenic pathway produces, among other fragments, the amyloid-β peptides which constitute the cytotoxic plaques characteristic of Alzheimer’s disease (AD) pathology (Mattson, 44; De Strooper and Annaert, 19). Conversely, the nonamyloidogenic pathway produces secreted APPα (sAPPα), the α-C-terminal fragment (α-CTF) and others (Mattson, 44; De Strooper and Annaert, 19). In addition to many reported physiological functions of holo-APP, the peptide fragments each have distinct roles in a variety of cellular processes occurring in the brain and other organs (Turner et al., 67). Numerous reports of sAPPα potentiating neurite outgrowth, preventing neuronal death and aiding in NSC proliferation confirm its neurotrophic and neuroprotective properties (Furukawa and Mattson, 23; Fu et al., 21; Copanaki et al., 16). Further, studies in isolated human NSCs suggest that sAPPα may promote astroglial cell-fate (Kwak et al., 33,b) via the IL-6/gp130 signaling pathway (Kwak et al., 35).

In light of the role of sAPPα in CNS development, and recent studies demonstrating elevations of sAPPα in the plasma of autistic children (Sokol et al., 61; Ray et al., 52), we hypothesized that autism patient subsets would exhibit elevations in brain sAPPα and mice designed to overexpress human sAPPα in brain tissues would reveal signs suggestive of gliosis and autism-like behavior.

## Materials and Methods

### Human Sample Preparation

Postmortem specimens from the insular cortex brain region of 8 normally developed controls and 6 autism patients (Table 1) were obtained from through the Autism Tissue Program from the National Institute of Child Health and Human Development Brain and Tissue Bank (NICHD, University of Maryland, Baltimore, MD). Approval for studies involving these specimens was granted by the institutional review board of the University of South Florida. Autism diagnoses were determined using the Autism Diagnostic Interview - Revised (ADI-R). Human brain samples were homogenized in 1X RIPA buffer (Cell Signaling Technology, Boston, MA) with 1% PMSF, and centrifuged at 14,000 rpm for 90 min at 4°C before storage at −80°C. Before use, samples were centrifuged at 14,000 rpm for 2 h at 4°C.

**Table 1:** Study Populations Characteristics

Characteristic	Autism	Control	*P*
*n*	6	8	
Age, mean (±SD)	7.4 (2.2)	7.2 (2.5)	0.46
Gender, *n* (% female)	1 (16.7)	3 (37.5)	0.22
Nonwhite, *n* (%)	3 (50)	3 (37.5)	0.34
Postmortem interval, mean hours (±SD)	19.3 (7.4)	17.9 (8.7)	0.39

### Enzyme-Linked Immunosorbent Assays

Human sAPPα expression in brain homogenates and cultured cells was quantified using a highly specific assay kit (IBL-America, Minneapolis, MN). Levels of sAPPα were measured for each sample in duplicate according to manufacturer’s instructions. Commercially available enzyme-linked immunosorbent assay (ELISA) kits were used for measurements of IL-6, IL-1β, IL-4, TNFα, and IFN-γ according to manufacturers’ instructions.

### Mice and Genotyping

TgsAPPα mice were generated at the H. Lee Moffitt Cancer Center Animal Core Facility (Tampa, FL) by standard pronuclear injection using a 1.8 kb genomic fragment transcribing hsAPP-α695 sub-cloned into a MoPrP.Xho vector (Bailey et al., 2012). Mice were housed in a 12-h light-dark cycle, and genotyped using quantitative real-time PCR. All tissue collection and experiments were conducted in accordance with institutional guidelines and were approved by the University of South Florida institutional animal care and use committee.

### Behavioral Tests

#### Open field

Spontaneous locomotor activity and anxiety in mice were assessed in an open field consisting of a 17 inch square arena with plastic walls and floor evenly illuminated by white light. Each mouse was placed in the center of the field and allowed to explore for 10 min. Experiments were video-recorded and total distance traveled as well as time spent in center field was measured using Ethovision behavior analysis software (Noldus Information Technology, Leesburg, VA).

#### Social interaction

Test mice were first habituated to the 22 × 15 inch rectangular, three-chambered polycarbonate box with divider walls containing doorways allowing access to each chamber for 10 min. Each of the two side chambers contained an empty wire cage (Galaxy Cup, Spectrum Diversified Designs, Streetsboro, OH) that was inverted and weighted down. After habituation, test mice were enclosed in the center and an unfamiliar mouse of the same strain and gender was enclosed inside one of the wire cages. The location for the “stranger” mouse was alternated between left and right sides of the box. Test mice were allowed 10 min to explore. Experiments were video-recorded and measurements of the time spent in each chamber during both phases of the task were measured and analyzed using Ethovision behavior analysis software (Noldus Information Technology).

### Mouse Brain Tissue Isolation and Preparation

Mice were anesthetized using gaseous isoflourane and transcardially perfused with cold 0.01 M PBS (pH 7.4). Brains were rapidly removed and sagittally bisected. Left hemispheres were separated into hippocampus, striatum, cerebellum and cortex regions and each region was homogenized in 1X lysis buffer (Cell Signaling Technology) with 1% PMSF (Sigma-Aldrich, St. Louis, MO), centrifuged at 14,000 rpm for 15 min and stored at −80°C. Right hemispheres were fixed overnight with 4% paraformaldehyde and cryoprotected in a graded series of 10, 20, and 30% sucrose solutions, each overnight at 4°C. Right hemispheres were then embedded in Neg50 frozen section medium (Richard-Allan Scientific, Kalamazoo, MI), and sectioned sagittally on a Microm HM 550 cryostat (Thermo Scientific, Richard-Allan Scientific, Kalamazoo, MI) at 25 μm thickness. Free-floating sections were preserved in PBS containing 100 mM sodium azide at 4°C or PBS with 30% glycerol and 30% ethylene glycol at −20°C.

### Immunohistochemistry

Sections were washed in PBS, blocked in 5% horse serum in PBS for 1 h at room temperature and incubated overnight at 4°C in blocking solution containing one of the following antibodies: mouse monoclonal anti-Aβ_1-17_ (6E10, 1:2,000, Covance Research Products, Emeryville, CA); mouse monoclonal anti-GFAP (1:500, Cell Signaling Technology) rabbit polyclonal anti-gp130 (1:200, Novus Biologicals, Littleton, CO). Sections were then washed and incubated for 1 h with biotinylated secondary antibody that was developed by the ABC kit (Vector Laboratories, Burlingame, CA) with 3,3 diaminobenzidine tetrahydrochloride (DAB, Vector Laboratories). Sections were mounted on Permafrost slides and dehydrated with 2 min serial emersions in 95 and 100% anhydrous ethanol and xylene. Some sections were counterstained with 0.1% cresyl violet (Sigma-Aldrich) for Nissl staining (Zhu et al., 71).

### Western Blotting

Brain homogenates and cell lysates were subjected to SDS-PAGE on 10% glycine gels with Tris-Glycine-SDS buffer (BioRad Laboratories, Hercules, CA) and transferred to 0.45 μm nitrocellulose membranes (Amersham Biosciences, Piscataway, NJ) in Tris-Glycine buffer. After blocking with 5% milk in 1X TBS, blots were incubated overnight at 4°C with either of the following primary antibodies: mouse monoclonal anti-Aβ_1-17_ (6E10, 1:1,000, Covance Research Products); mouse glial fibrillary acidic protein (GFAP) (1:1,000, Cell Signaling Technology); mouse monoclonal anti-β-tubulin (1:1,000, Stem Cell Technologies, Tukwila, WA); rabbit polyclonal anti-APP C terminus (pAb 396, 1:1,000, kindly provided by S. Gandy and H. Steiner); rabbit polyclonal anti-Notch1 (1:1,000, Epitomics, Burlingame, CA); rabbit polyclonal anti-Notch 1 intercelluar domain (NICD, activated Notch 1) (1:500, Abcam, Cambridge, MA); rabbit polyclonal anti-gp130 (1:1,000, Merck Millipore, Darmstadt, Germany); mouse monoclonal anti-β-actin (1:4,000, Sigma-Aldrich). After washing with ddH_2_O, blots were incubated for 1 h at room temperature with one of the following horseradish peroxidase-conjugated secondary antibodies: horse anti-mouse IgG-HRP linked (1:1,000, Cell Signaling Technology); goat anti-rabbit IgG-HRP linked (1:5,000, Cell Signaling Technology). Blots were developed using Supersignal West Femto Maximum Sensitivity Substrate (Thermo Fisher Scientific).

### Cell Culture

Cortical primary neurons were isolated from E14 embryos of heterozygous-bred TgsAPPα dams. TgsAPPα and wild-type (WT) littermate mouse embryonic brain tissues were mechanically dissociated. Both primary cortical neurons and commercially available murine neurospheres (Stem Cell Technologies, Tukwila, WA) were cultured in suspension in DMEM/F12 (Invitrogen, Camarillo, CA) containing B27 (Invitrogen), 20 ng/mL human epidermal growth factor (hEGF), and 10 ng/mL fibroblast growth factor (fGF) at 37°C in 5% CO_2_. Primary neuron cultures from each embryo remained separate and genotypes were identified from embryo tails using real time PCR (Bailey et al., 2012). For differentiation, neurospheres were mechanically dissociated and filtered with a 40 μm cell strainer into single-cell suspensions in DMEM/F12 containing B27, 20 ng/mL hEGF, 10 ng/mL fGF and 10% fetal bovine serum and plated in 24-well plates (Fisher Scientific) at a concentration of 1x10^5^ cells per well and incubated at 37°C in 5% CO_2_. Human HEK293-expressed human sAPPα (hsAPPα) was generated and purified as previously described (Barger et al., 8; Furukawa et al., 22). N2a (murine neuroblastoma) cells (ATCC, Manassas, VA) were grown in complete EMEM supplemented with 10% fetal calf serum. Cells were plated in 24-well collagen coating culture plates at a density of 1x10^5^ cells per well. After overnight incubation, N2a cells were incubated in neurobasal media supplemented with 3 mM dibutyryl cAMP in preparation for treatment. N2a cells stably overexpressing human sAPPα (N2a/sAPPα cells, named 6-1 cell clone) were generated via liposomal delivery using Lipofectamine 2000 (Invitrogen) of a plasmid (pcDNA3.1-sAPP-α695) containing a human sAPPα cDNA coding sequence based on the predicted cleavage of the 695 aa isoform of APP (a generous gift from Dr. Steven Barger, University of Arkansas) into N2a cells followed by G418 (400 μg/mL) selection.

### Immunocytochemistry

After 7 days in culture, neurospheres were gently and mechanically triturated into single-cell suspensions, plated on chamber slides at a concentration of 20,000 cells per well and incubated for 3–7 days at 37°C in 5% CO_2_. Cells were fixed with 4% paraformaldehyde (Fisher Scientific) in phosphate-buffered saline (PBS) for 20 min at room temperature, permeabilized with 0.2% Triton X-100 for 5 min at room temperature, blocked in 5% horse serum for 1 h at room temperature, and incubated overnight at 4°C in 5% horse serum containing one of the following primary antibodies: monoclonal anti-β-tubulin (1:1,000, Stem Cell Technologies), mouse monoclonal anti-GFAP (1:500, Cell Signaling Technology). Cells were then incubated for 30 min at room temperature with fluorescein-conjugated AlexaFluor 488, AlexaFluor 594, and AlexaFluor 555 secondary antibodies (Fluorescein-conjugated IgGs, Invitrogen) at a dilution of 1:200. Images were acquired on an Olympus FV1000 Confocal microscope.

### Statistical Analysis

Statistical differences between genotype groups were determined using one-way analysis of variance (ANOVA) for multiple comparisons. Other statistical differences were determined using the Student’s *t*-test. Statistical analysis for behavioral experiments was performed using GraphPad5 Analysis software. Analyses were performed on Microsoft Excel software.

## Results

### Elevated sAPP-α and α-CTF Levels in Brain Tissues From Autistic Children

**Figure 1:**
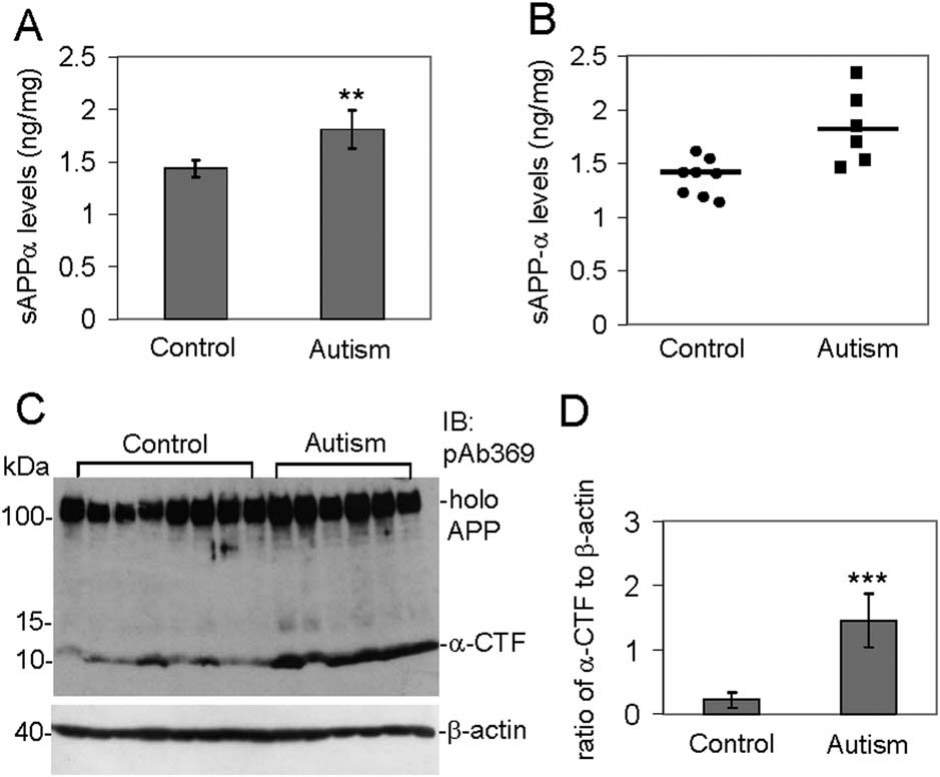
Levels of human sAPPα (hsAPPα) and α-CTF are elevated in brain tissues from autistic children. A, B: Human sAPPα (hsAPPα) levels in brain homogenates from 6 autistic and 8 age-matched typically developed children (see Table1). A: Data are represented as mean ± sd or (B) dot-plot (hsAPPα ng/mg total protein). C: Western blot (WB) analysis shows increased α-CTF levels in autistic children compared to controls. D: Densitometry analysis showing band density ratios of α-CTF to β-actin. This α-CTF band was further identified by WB using Aβ_17–24_ antibody, 4G8 (specifically selective for human α-CTF) (data not shown). **P < 0.01; ***P < 0.001.

Previous studies report elevated levels of sAPPα in the plasma of children with severe autism (Ray et al., 52). Since sAPPα is an established neurotrophic factor (Mucke et al., 48; Turner et al., 67), concentrations in autistic patient brains may be affected. A commercially available human sAPPα ELISA kit was used to measure the sAPPα concentrations in brain homogenates from autistic patients and controls. Results show that autistic patient brains contain a significantly increased level of sAPPα compared to the mean level found in the brains of normally developed controls (Fig. 1A). The distributions of individual patient sAPPα levels around the means show that 4 of the 6 autistic patients had sAPPα levels greater than the mean sAPPα levels of the controls (Fig. 1B).

In light of the nonamyloidogenic processing pathway, increased levels of sAPPα in the brain should convey a corresponding increase in the levels of the APP α-C-terminal fragment (α-CTF). Patient brain tissue samples were analyzed by western blot using the polyclonal antibody 396 which binds the APP C-terminal fragment. The results, along with densitometric analysis, show significantly increased expression of the α-CTF fragment in autistic patient brains compared to normal control brains (Fig. 1C,D). This data suggests that subsets of autistic patients have elevated brain levels of the sAPPα and α-CTF fragments of APP, signifying a greater inclination towards the non-amyloidogenic pathway in patient brains and a potential role for the associated fragments in the pathophysiology of autism.

### TgsAPPα Mice Have Elevated hsAPPα Levels in the Brain

**Figure 2:**
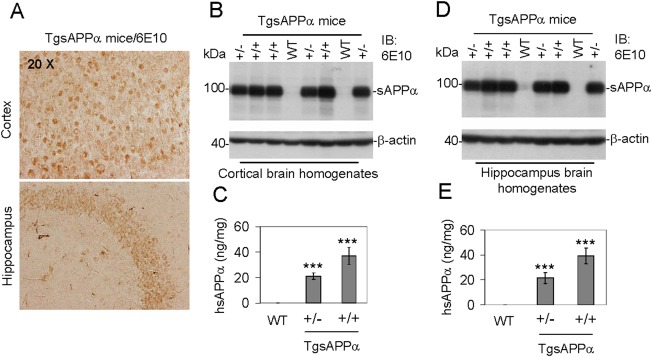
Brain expression of human sAPPα (hsAPPα) in the TgsAPPα mouse. A: Brain sections from homozygous TgsAPPα mice stained with Aβ_1–17_ antibody 6E10. Cortical and hippocampal brain homogenates were prepared from a single family of 3-month-old TgsAPPα mice and WT (WT) littermates (TgsAPPα^+/+^, *n* = 3; TgsAPPα^+/−^, *n* = 3; WT mice, *n* = 2). The hsAPPα levels in cortical (B, C) and hippocampal (D, E) brain homogenates were determined by WB analysis and ELISA. For C and E, data are presented as mean ± sd (sAPPα ng/mg total protein). Similar results were also observed in littermates from three different families (data not shown). ***P < 0.001.

To identify the potential role of elevated sAPPα levels on neurodevelopment, our group has studied a transgenic mouse model that overexpresses human sAPPα in the brain (Bailey et al., 2012). Brain sections and homogenates from a set of 3-month-old TgsAPPα littermates were studied by immunohistochemistry and Western blotting techniques using the 6E10 antibody which specifically binds human APP at the Aβ_1–17_ region. Homozygous TgsAPPα (TgsAPPα^+/+^) mouse brains stained positive for 6E10 in both the cortex and the hippocampus, confirming the expression of the human sAPPα fragment in these brain regions (Fig. 2A). Western blot analysis and ELISA measurements further corroborated the expression of the protein fragment in TgsAPPα mice (Fig. 2B–E), demonstrating its absence in WT littermates and genotype-dependent levels of fragment expression in both cortex (Fig. 2B,C) and hippocampus (Fig. 2D,E) regions. These studies confirm the overexpression of hsAPPα in TgsAPPα mice.

### Impaired Social Behavior and Hypoactivity in TgsAPPα Mice

Social interaction deficits, increased anxiety and hypoactivity are characteristic behaviors of autism patients. To examine social functioning and motor activity in our transgenic mice, we used mouse behavioral tests for open field and social interaction (Crawley 17).

**Figure 3:**
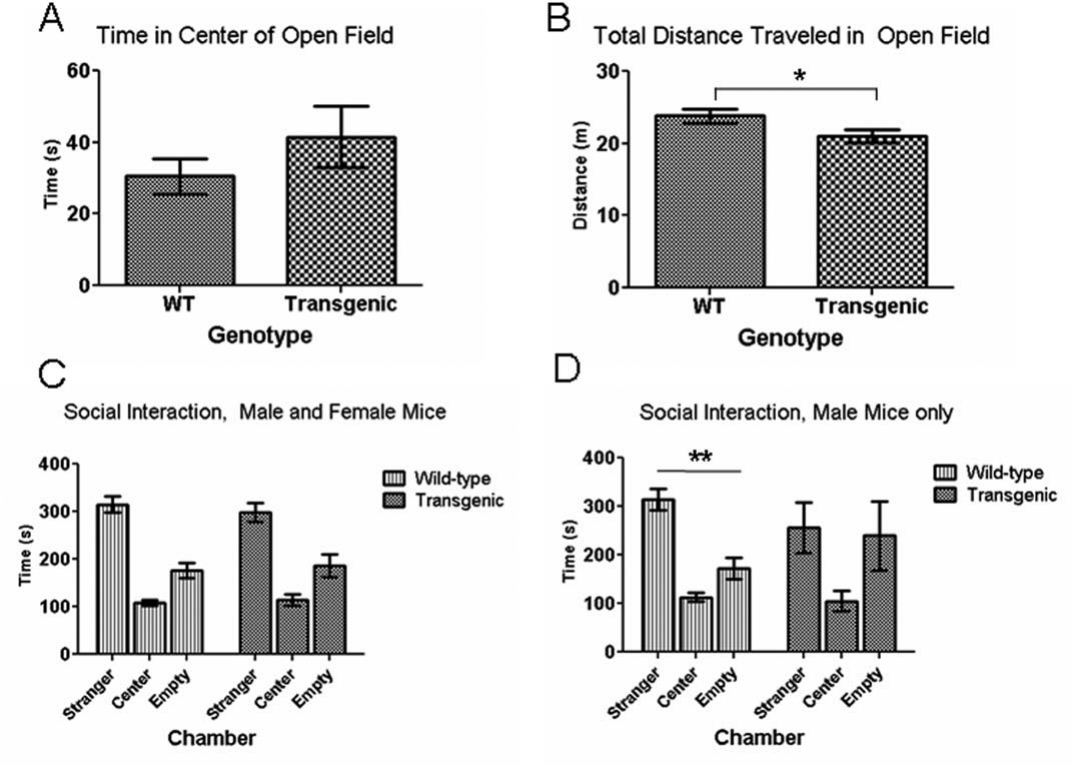
TgsAPPα mice exhibit hypoactivity and social impairment. A, B: Anxiety and locomotor activity were observed in 3-month-old TgsAPPα mice (*n* = 13) and WT littermates (*n* = 12). There was no significant difference between groups in time spent in the center (A). Transgenic mice traveled significantly less distance compared to WT littermates (B). C, D: Mice were also subjected to social interaction behavior testing. Mice from groups containing both males and females spent significantly more time in the chamber containing an unfamiliar mouse (stranger) compared to a chamber with an empty wire cage. A comparison of only males in each group showed that, for TgsAPPα male mice, there was no difference between the times spent in the empty chamber (Empty) and the stranger chamber (*P* > 0.05), while littermate control males spent significantly more time in the stranger chamber (**P < 0.01).

In the open field task, mice were placed in an open box for 10 min and observed for locomotor activity and time spent in the center of the field. There was no significant difference between groups in time spent in the center (Fig. 3A); however, transgenic mice traveled significantly less distance in the apparatus than WT littermate controls (Fig. 3B), suggesting hypoactivity.

For the social interaction experiment, test mice were observed for 10 min in a 3-chambered apparatus containing an empty wire cage on one end and a wire cage enclosing a stranger mouse on the other end. Whereas WT mice (*n* = 5) spent significantly more time in the chamber containing the stranger mouse than in the chamber with the empty wire cage, there was no significant difference in the periods of time that TgsAPPα mice (*n* = 4) spent in each of these chambers (Fig. 3C,D). Ratios of time spent with stranger/time spent with empty wire cage showed a decrease in time spent with stranger mice by TgsAPPα mice compared to WT littermates. This finding demonstrates a decreased preference for sociability in TgsAPPα mice compared to WT controls.

### Increased GFAP Expression in Brains of Adult TgsAPPα Mice

**Figure 4:**
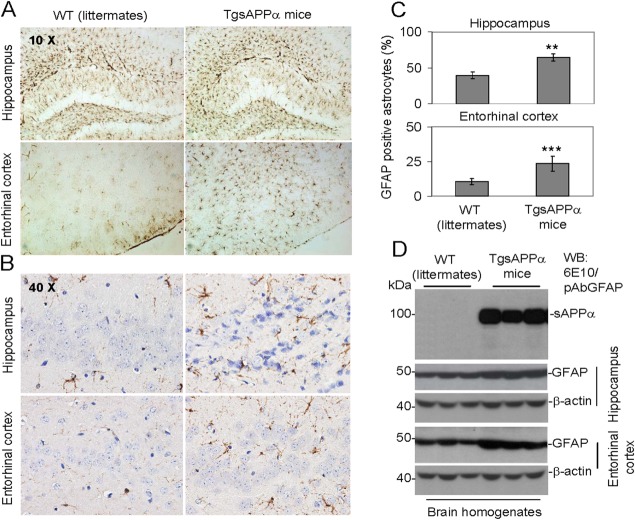
Increased GFAP expression in brains of adult TgsAPPα mice. A, B: Brain sections from 3-month-old TgsAPPα^+/+^ mice and WT littermates were stained with an anti-GFAP antibody (brown). C: Significant increases in the percentage of GFAP immunoreactive astrocytes from TgsAPPα mouse and WT littermate brain sections from hippocampus (top) and entorhinal cortex (bottom) regions (*n* = 4♀/4♂, per group). D: Entorhinal cortical and hippocampal homogenates subjected to WB analysis of GFAP reveal increased GFAP expression in both regions from TgsAPPα mice. Similar results also observed in 1- and 6-month-old TgsAPPα mice (data not shown). Data are presented as mean ± sd. **P < 0.01; ***P < 0.001.

An association between APP and increased GFAP expression in the brain has been firmly established in studies on patients suffering from AD and Downs Syndrome (DS) (Jorgensen et al., 28; Sugaya et al., 65). To find out whether the overexpression of sAPPα was associated with an increase in this astrocyte marker, brain sections from 3-month-old TgsAPPα mice and WT controls were subjected to immunohistochemical staining with GFAP (Fig. 4A). Other brain sections from these mice were double stained with GFAP and Nissl, which identifies cell bodies and delineates morphology in brain tissues (Fig. 4B). Image analysis and quantification of GFAP staining show that there are significantly more GFAP-positive cells in the hippocampus and entorhinal cortex of TgsAPPα mice compared to WT controls (Fig. 4C). Immunohistochemistry findings were verified by Western blot analysis on cortical and hippocampal brain homogenates and, as expected, significantly increased GFAP expression was found in TgsAPPα mice in both regions compared to WT controls (Fig. 4D). This GFAP increase is also seen in 1-month-old and 6-month-old TgsAPPα mice compared to WT controls (data not shown).

### Increased Glial Differentiation in TgsAPPα Derived Murine Neuronal Stem Cells

**Figure 5:**
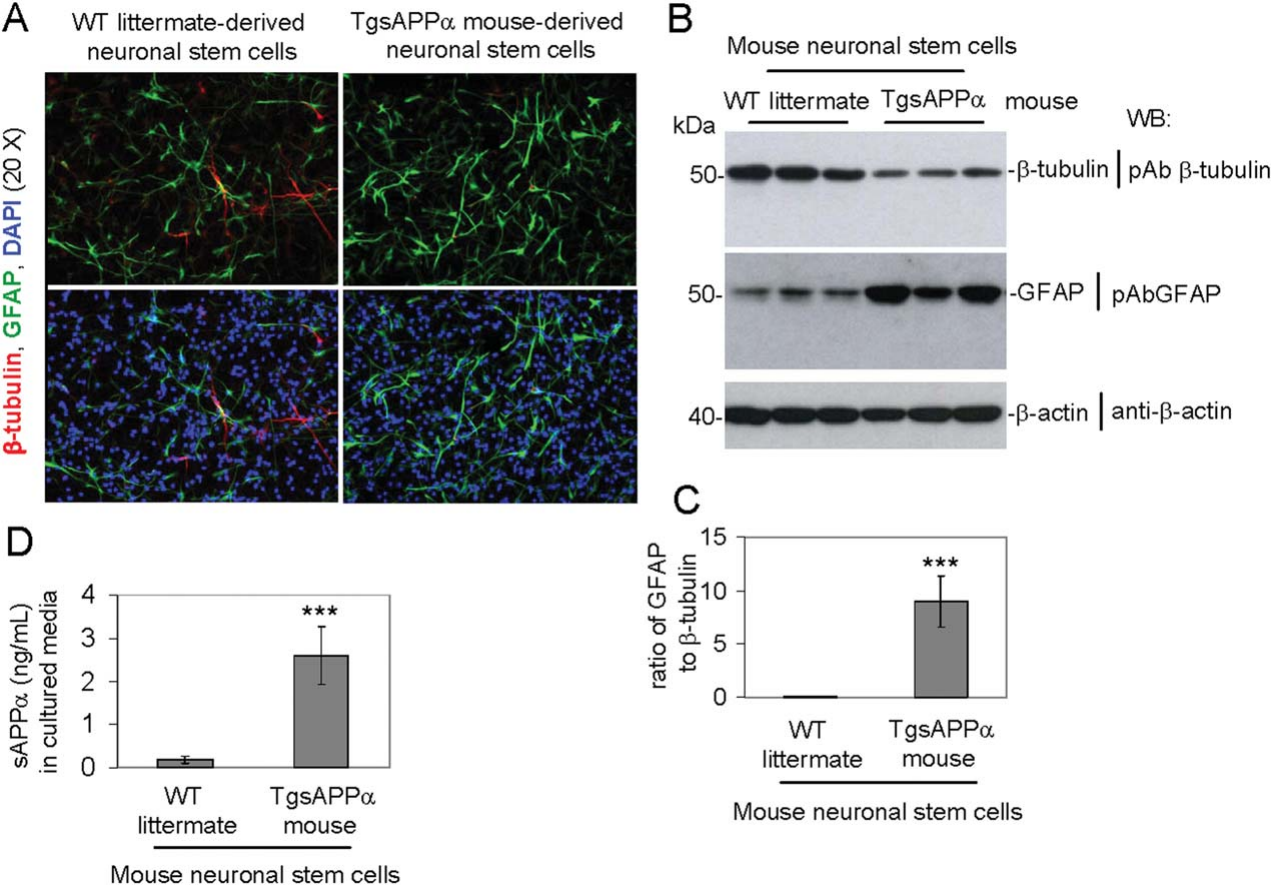
Suppressed β-tubulin III and elevated GFAP in TgsAPPα-derived murine NSCs. A: Representative photomicrographs of WT littermate and TgsAPPα mouse-derived NSCs under differentiating culture conditions (Day 3) showing increased GFAP (green) and decreased β-tubulin III (red) expression by immunofluorescence (IF). B: WB analysis of GFAP and β-tubulin III expression in both NSCs. C: Densitometric analysis revealed increased ratio of GFAP to β-tubulin III in TgsAPPα mouse-derived NSCs compared to WT littermate control. Data presented as mean ± sd. D: Relative concentrations of sAPPα secreted by WT littermate and TgsAPPα mouse-derived NSCs measured by ELISA and expressed as ng of sAPPα/mL media. TgsAPPα mouse NSCs secrete significantly greater concentrations of sAPPα than WT. These results are representative of three independent experiments with each condition triplicated. ***P < 0.001.

Murine NSCs derived from TgsAPPα and WT E14 embryos were cultured under differentiating conditions for 3 days. Using immunofluorescence techniques, these differentiated stem cells were fixed and stained with antibodies against neuronal marker β-tubulin III and GFAP. Compared to murine NSCs from WT embryos, there was less β-tubulin III expression and greater GFAP expression in murine NSCs derived from TgsAPPα embryos (Fig. 5A). Immunofluorescence findings were verified by Western blot analysis on cell lysates prepared from mouse NSCs (Fig. 5B). As expected, significantly increased GFAP expression was found in TgsAPPα mouse-derived NSCs (Fig. 5C). The levels of human sAPPα (hsAPPα) secreted into the media by the murine NSCs from both groups of mice were measured using a commercially available ELISA kit. The ELISA results confirmed that the hsAPPα transgene is expressed in murine NSCs from TgsAPPα embryos and that the protein fragment is secreted from these murine NSCs (Fig. 5D). Overall, these results support the association between sAPPα and increased GFAP expression *in vivo*.

### Increased Glial Differentiation in Wild-Type Murine NSCs Treated With Recombinant sAPPα

**Figure 6:**
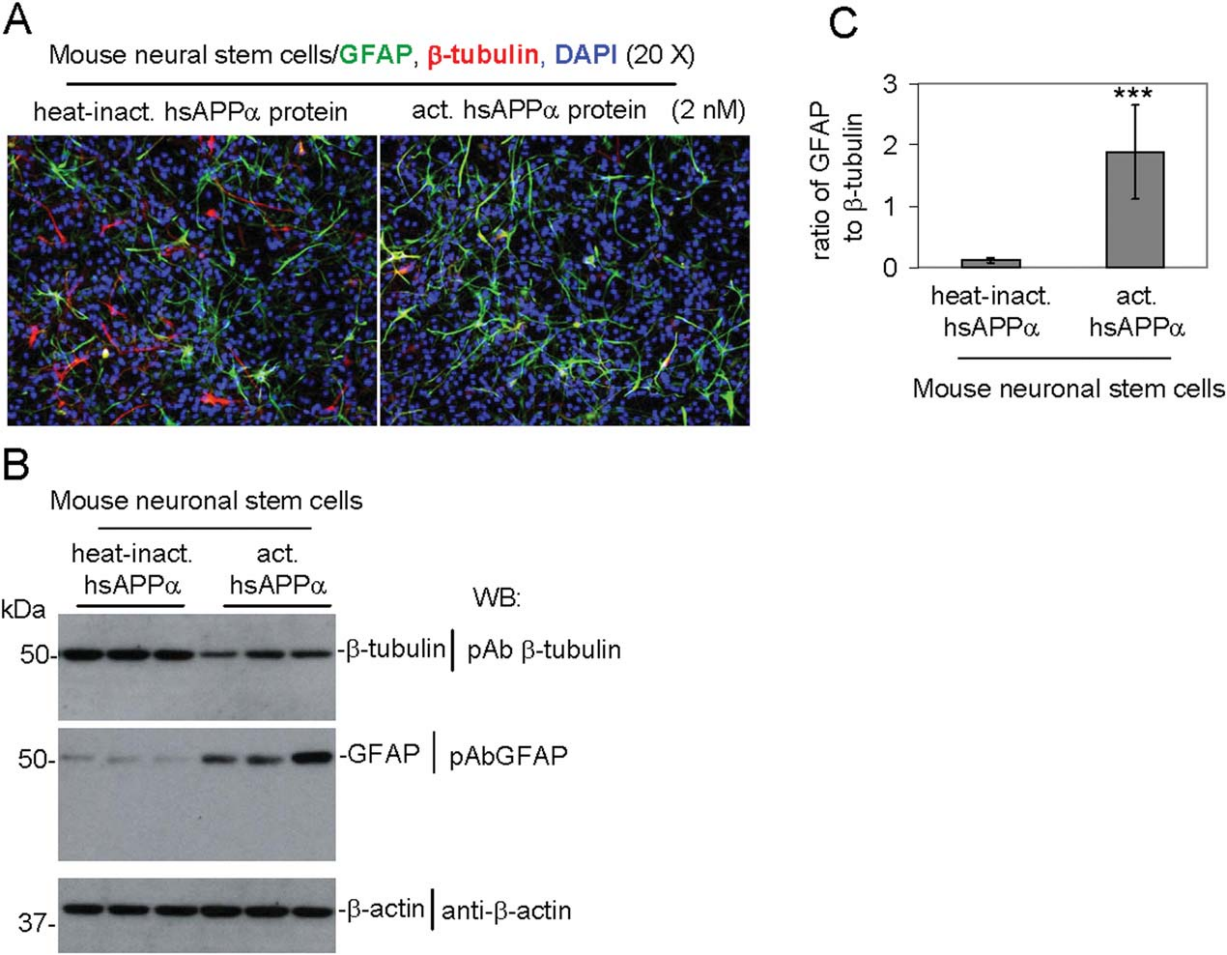
Suppressed β-tubulin III and elevated GFAP in WT murine NSCs treated with recombinant human sAPPα (rhsAPPα). A: Representative photomicrographs of murine NSCs treated with 2 nM rhsAPPα protein under differentiating culture conditions (Day 5) revealed enhanced GFAP (green) and suppressed β-tubulin III (red) expression by IF. B: WB analysis of GFAP and β-tubulin III after rhsAPPα treatment of NSCs. C: Densitometry analysis showed significantly increased ratio of GFAP to β-tubulin III in rhsAPPα treated NSCs compared with heat-inactivated hsAPPα (heat inact.). Data presented as mean ± sd. These results are representative of three independent experiments with triplicates for each condition. ***P < 0.001.

Murine NSCs were treated with 2 nM concentrations of recombinant human sAPPα (rhsAPPα) under differentiating conditions composed of 2% fetal bovine serum (FBS) in complete culture medium containing growth factors. In three independent experiments using immunofluorescence staining after 5 days of culture, enhanced GFAP (green) and suppressed β-tubulin III (red) expression were observed under these conditions in murine NSCs treated with rhsAPPα compared to NSCs treated with heat-inactivated rhsAPPα (*n* = 3 for each culture condition) (Fig. 6A). Western blot analysis further confirmed this observation (Fig. 6B,C). These results further suggest that excess sAPPα may promote glial cell fate in isolated murine NSCs.

### Increased Expression of IL-6, NICD, and gp130 Correlated With Elevated sAPPα Levels

**Figure 7:**
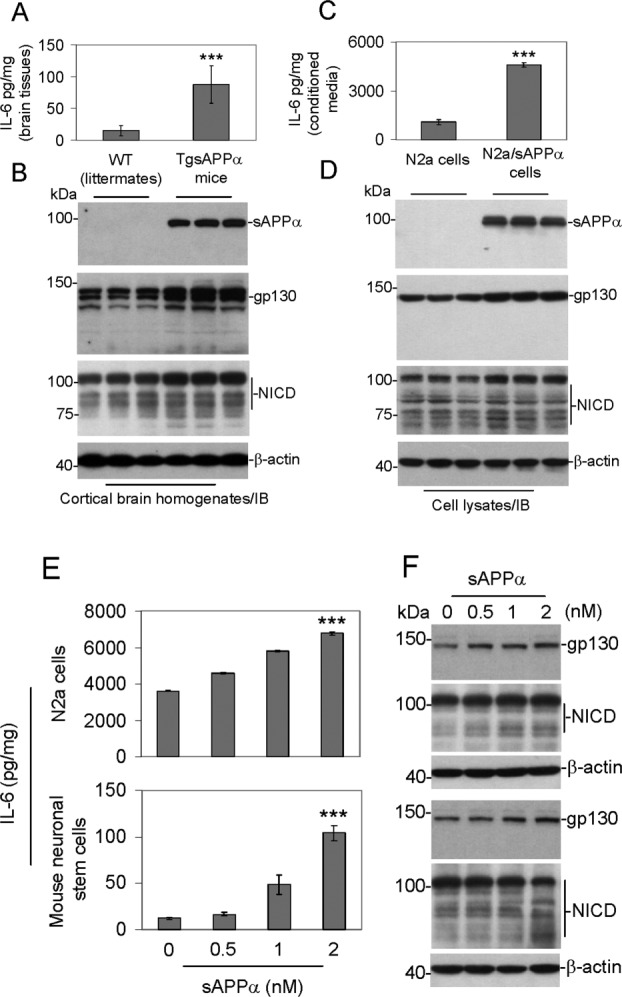
Increased expression of IL-6, NICD, and gp130 correlated with elevated sAPPα levels. A: Cortical brain homogenates were prepared from 3-month-old TgsAPPα mice and WT littermates. (*n* = 4♂) and subjected to IL-6 ELISA (mean ± sd of pg of IL-6/mg total protein). IL-6 production was enhanced in cortical tissues from TgsAPPα mice. B: WB analysis of these tissues for sAPPα (6E10), gp130, NICD, and β-actin. C: Conditioned media collected after 18 h from N2a cells overexpressing human sAPPα (6-1 cell clone) show enhanced IL-6 production by ELISA compared to N2a cells. Data presented as mean ± SD (pg of IL-6/mg protein) from three independent experiments, with three replicates per group. D: WB analysis of these cells for sAPPα, gp130, NICD, and β-actin. WT N2a cells (E, top panel) or murine NSCs (E, bottom panel) were treated with sAPPα at doses indicated for 24 h and subjected to IL-6 ELISA revealing enhanced levels of IL-6 in sAPPα treatment groups. IL-1β, IL-4, TNFα, and IFN-γ were undetectable (data not shown). F: In parallel, the cell lysates were subjected to WB analysis using gp130 and NICD antibodies. Data presented as mean ± sd (pg of IL-6 per mg total protein from three independent experiments, three replicates per group). **P < 0.01; ***P < 0.001.

Standard mechanisms of action by which sAPPα achieves its effects in the brain are still yet to be determined. With these experiments, we aimed to identify possible signaling pathways involved in sAPPα function. Previous studies implicate the IL-6/gp130 pathway and the activity of Notch1 intracellular domain (NICD, activated Notch1) in glial differentiation of NSCs (Rodriguez-Rivera et al., 55; Kwak et al., 35). IL-6 concentrations in cortical brain homogenates from 3-month-old WT (*n* = 4♂) and TgsAPPα (*n* = 4♂) mice were measured by ELISA. TgsAPPα mice demonstrated a significantly increased mean concentration of IL-6 per milligram of protein than WT littermates (Fig. 7A). Brain homogenates were also subjected to Western blot analysis using antibodies against human sAPPα, gp130, NICD, and β-actin. The blots exhibited greater protein expression of gp130 and NICD in the TgsAPPα mice, which verifiably express hsAPPα, compared to WT littermates (Fig. 7B).

To determine whether this effect is specifically due to the presence of hsAPPα, we transfected N2a neuroblastoma cells with the hsAPPα gene to create a cell line, named clone 6-1, which overexpresses the hsAPPα protein fragment. Conditioned media from triplicate cultures of each of these two cell lines were collected 18 h after plating (without treatment) and subjected to IL-6 ELISA. The 6-1 cells secreted a significantly higher level of IL-6 than N2a cells under normal culture conditions (Fig. 7C). Lysates from each cell line were analyzed by Western blot using antibodies against hsAPPα, gp130, activated Notch1, and β-actin. Results confirm the successful transfection of the hsAPPα fragment in the 6-1 line and show increased expression of gp130 and NICD in this cell line compared to the WT neuroblastoma line (Fig. 7D).

Finally, N2a cells and murine NSCs were treated with 0, 0.5, 1, and 2 nM doses of recombinant hsAPPα (rhsAPPα) for 24 h. IL-6 concentrations secreted into the media were measured by ELISA and the cell lysates were subjected to Western blot analysis using antibodies against gp130, activated Notch1, and β-actin. Although the murine NSCs secreted notably less IL-6 than the N2a cells, treatment with rhsAPPα induced both types of cells to secrete increased levels of IL-6 in a dose-dependent manner (Fig. 7E). Dose-dependent increases in gp130 and activated Notch1 were also evident in cell lysates after treatment with rhsAPPα (Fig. 7F).

**Figure 8:**
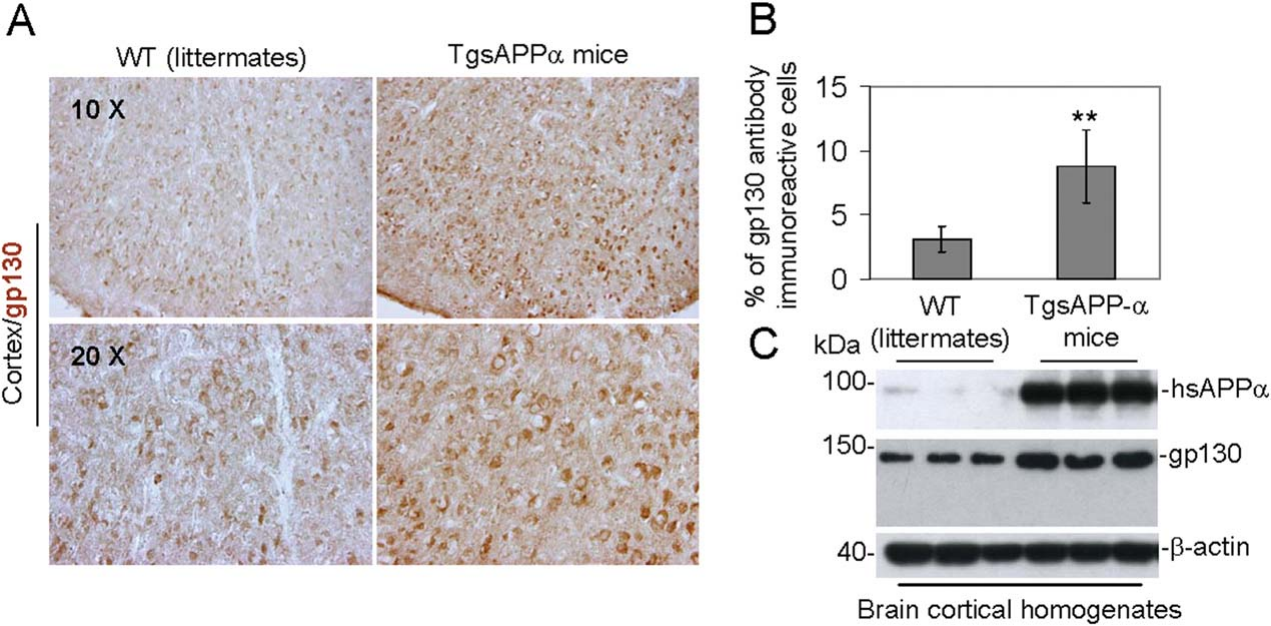
TgsAPPα mice show a marked increase in gp130 levels in entorhinal cortex. A: Representative brain sections from 3-month-old TgsAPPα mice and WT littermates (*n* = 4♀/4♂, per group) stained with gp130 antibody (brown signal). B: Enhanced percentages of gp130 immunoreactivity in the TgsAPPα group compared to WT littermate controls. C: WB analysis for gp130 from cortical tissues showed increased gp130 expression in mice overexpressing hsAPPα. Similar results were also observed in TgsAPPα mice at 1 and 6 months of age (data not shown). **P < 0.01.

In addition, we observed the cortical expression of gp130 in TgsAPPα mice and WT controls by immunohistochemistry. TgsAPPα mice exhibit increased gp130-positive staining compared to WT littermates (Fig. 8A,B). As further support, mouse brain homogenates were prepared from dissected cortical tissues and subjected to Western blot analysis. Notably, these results confirmed the increased expression of gp130 in the cortical region (Fig. 8C).

## Discussion

This work reports the discovery of elevated levels of sAPPα and α-CTF in the insular cortex of autism patients and suggests increased GFAP expression-associated hypoactivity and social deficits in mice designed to over-express hsAPP in brain tissues. The work further reveals that the observed GFAP upregulation is correlated with elevations in IL-6, gp130, and Notch1. These observations support our hypothesis that subsets of patients with autism exhibit elevations in CNS sAPPα and mice generated to mimic this observation display markers suggestive of gliosis and autism-like behavior.

Since the neurotrophic and neuroprotective functions of the sAPPα fragment are generally accepted, previous studies revealing the presence of sAPPα in autism patient plasma raise questions about the association between the APP fragment and autism pathophysiology (Sokol et al., 61; Bailey et al., 2012). We discovered, in this study, that autism patients exhibit elevated levels of sAPPα as well as notable increases in α-CTF compared to controls in a portion of the insular cortex (Fig. 1) which is part of the gray matter in the CNS. The insular cortex was selected because it is part of the neocortex that shows neurobiological abnormalities in some autistic populations and appears to be involved in language and attention (Bailey et al., 4; Binstock, 12). The initial study proposing this unconventional association mentioned that the presence of the sAPPα fragment could be evidence of heightened α-secretase processing (Sokol et al., 61); and our findings, particularly the dramatic increase in α-CTF levels, support this inference.

APP is in fact upregulated in the brain in response to neural injury, but, as previously mentioned, sAPPα plays a neuroprotective role (Mattson et al., 45). As Table1 shows, several of the samples from both control and autistic cases involve causes of death that could impact brain APP levels. Some of the individual causes of death occur in both groups of cases, thereby alleviating a potential confounding variable. For example, a comparison of the levels of α-CTF in control cases of drowning with the α-CTF level in the autism case of drowning shows increases in α-CTF expression in the control cases of drowning compared to control cases with other causes of death. However, there is an even greater increase in α-CTF expression within the brain sample from the autistic case of drowning compared to the control cases.

Our data confirms that the TgsAPPα mouse we generated exhibits greater expression of hsAPPα in the cortex and hippocampus regions of the brain compared to WT littermate controls. In light of our observations of the autistic insular cortex, the TgsAPPα mice mimic the human condition of elevated cortical sAPPα levels. We have previously reported that these mice also demonstrate high sAPPα levels in the plasma (Bailey et al., 2012), which also mimics the original finding in autism patient plasma (Sokol et al., 61). It is worth noting that hsAPPα in heterozygous TgsAPPα mice is expressed at over 10 times the sAPPα levels we observed in the human condition, and homozygous mice have over 20 times greater hsAPPα expression compared to patients studied here. This exaggeration of sAPPα overexpression in the model represents a significant limitation in the translation of the results to the human condition.

Additionally, TgsAPPα mice demonstrate behavioral impairments that simulate autistic behaviors in humans. In addition to impaired social interaction, which is a cardinal autistic behavioral phenotype, patients have also demonstrated hypoactivity (Gillberg and Billstedt, 25; Maestro et al., 41; Receveur et al., 53). TgsAPPα mice exhibit reduced preference for social interaction, indicating impaired sociability (Fig. 3A). Further, in the open field task, TgsAPPα mice demonstrated hypoactive exploratory behavior (Fig. 3C,D). Altogether, the neuropathological and behavioral features of TgsAPPα mice are suggestive of components of an autism phenotype, however further studies are required to substantiate sAPPα’s direct causation.

Evidence of increased GFAP and gliosis in autism patients has been demonstrated in several studies (Bailey et al., 4; Sabaratnam, 56; Casanova, 14). One study shows that the level of GFAP in the CSF of autism patients was at almost three times the level normally developed patients (Ahlsen et al., 1). Vargas et al. and Fatemi et al. report increased GFAP reactions in different regions of autistic patient postmortem brains, including the middle frontal gyrus and the superior frontal cortex (Laurence and Fatemi, 38; Vargas et al., 68). These observations in autism patients may be related to the longstanding theory that astrocytes participate in the brain’s immune response and indicate tissue damage (Casanova, 14). Adult TgsAPPα mice demonstrate increased GFAP expression in the entorhinal cortex and hippocampus regions compared to WT littermate controls (Fig. 4). These results suggest a possible role for the elevated levels of sAPPα and increased GFAP expression observed in postmortem autism brains.

There is minimal emphasis in the literature on sAPPα function in other brain cells besides neurons. Its trophic and protective effects on neurons have been well-documented (Masliah et al., 43; Milward et al., 47; Mattson et al., 46; Roch et al., 54; Smith-Swintosky et al., 59; Luo et al., 40). Evidence exists supporting sAPPα activity on microglial cells, stimulating the release of interleukin-1 (Li et al., 39), glutamate and markers of inflammation (Barger and Harmon, 9; Barger and Basile, 7). The cellular function of sAPPα in astrocytes was introduced with reports that treatment of human NSCs with recombinant sAPPα caused increased astrogliogenesis *in vitro* (Kwak et al., 33). More recently, sAPPα has been proven to increase subgranular zone (SGZ)-derived neural progenitor cell (NPC) proliferation in culture, and treatment of these cells with sAPPα increased NPC differentiation into astroglial cells (Baratchi et al., 2011).

We observed increased GFAP expression and decreased β-tubulin III expression by primary murine NSCs derived from TgsAPPα mice (Fig. 5). We also detected dose-dependent increases in GFAP-positive cells differentiated from WT murine NSCs treated with rhsAPPα (Fig. 6), suggesting that in cell cultures sAPPα could promote glial cell fate. Despite these findings it is likely that upregulation of α-CTF itself (Fig. 1) has effects on APP and its binding partners beyond those of sAPPα. Interestingly, it is not known whether α-CTF and sAPPα can bind together and form a heterodimeric receptor as in the case of Notch1 and other similar receptors (Blaumueller et al., 1997; Furukawa et al., 22; Van Nostrand et al., 2002; Shaked et al., 2006; Chen et al., 2006; Kedikian et al., 2010; Isbert et al., 2012). Hence the properties of α-CTF in subsets of individuals with autism remain elusive.

Several binding partners for sAPPα have been identified, such as apolipoprotein E (Barger and Harmon, 9), the class A scavenger receptor (Santiago-Garcia et al., 57), and others (Kounnas et al., 31; Knauer et al., 30; Das et al., 18), providing a number of candidates that may be responsible for its cellular activities. However, none of these proteins or receptors is involved in glial differentiation. IL-6 family proteins promote the differentiation of NSCs into astrocytes and inhibit neurogenesis through activation of the JAK-STAT pathway (Bonni et al., 13). The binding of leukemia inhibitory factor (LIF) ligand to its receptor recruits membrane gp130 to form a complex that triggers the JAK-STAT pathway, leading to gliogenesis in NSCs (Taga and Fukuda, 66). Additionally, Notch1 inhibits neuronal cell fate and promotes glial differentiation of NSCs via its intracellular domain (Wang and Barres, 69; Gaiano and Fishell, 24). In particular, Notch1 guides cells that were already destined to become glia towards an astrocytic rather than oligodendrocytic cell fate (Grandbarbe et al., 26; Lasky and Wu, 37). Sugaya et al. showed that sAPPα induces glial differentiation of human NSCs by activating the IL-6/gp130 pathway (Kwak et al., 35). This group also reported that Notch1 signaling is involved in sAPPα-induced glial differentiation of NSCs (Kwak et al., 36). Our findings confirm these sAPPα mechanisms of action in murine NSCs. We observe increased expression of IL-6 and gp130 in NSCs from TgsAPPα mice and N2A cells treated with conditioned medium from NSCs obtained from TgsAPPα mouse NSC culture. Moreover, our observation of increased NICD expression after these experiments suggests that sAPPα operates through the Notch1 pathway (Fig. 7). The sAPPα fragment may not be directly binding to the receptors that trigger either the IL-6 or Notch1 pathways; however, these findings imply that other sAPPα binding partners exist. Velasco et al. reported that stimulation of glial differentiation in rat NSCs via the Notch1 pathway is more potent than stimulation via the IL-6/gp130 activated pathway (Rodriguez-Rivera et al., 55). Our results suggest that sAPPα can promote glial cell fate of murine neural cells.

In addition to its well-established function in glial differentiation during development, gp130 participates in the regulation of serotonergic gene expression in the mouse brain (Kulikov et al., 32) and, along with other constituents of the IL-6 pathway, is activated in the astrocytic response to traumatic brain injury (Oliva et al., 51). Kirsch et al. previously reported that the IL-6/gp130 pathway regulates the astrocytic response and axonal sprouting of neurons after entorhinal cortex lesion in adult rats (Xia et al., 70). Here, we report that adult TgsAPPα mice demonstrated marked increases of gp130 in the entorhinal cortex by immunohistochemistry (Fig. 8). This suggests that the overexpression of sAPPα in the brain is associated with continually increased presence of gp130, even after development. In light of the aforementioned results (Fig. 4), this gp130 increase may correlate with the GFAP increase we observed in TgsAPPα mice; however direct causation is lacking. Whether excess sAPPα may potentially be impacting gliosis and abnormal brain development through upregulation of gp130 and consequently gp130-related pathways such as Notch1 and LIF in some individuals with autism remains to be determined.

In summary, we have shown that subsets of patients with autism exhibit elevations in sAPPα and α-CTF in the insular cortex compared to normally developed children. Mice generated to mimic this observation show abnormal social behavior and hypoactivity, increased GFAP expression, and decreased β-tubulin III with associated elevations in IL-6, gp130, and Notch1. NSCs derived from sAPPα overexpressing mice, as well as N2a cells treated with rhsAPPα displayed increased markers suggestive of glial cell fate. Gliosis during critical windows of development may contribute to dysregulated synaptic pruning, maintenance or function, leading to aberrant synaptic connections and components of the autistic phenotype.
